# Supplementary material for: An Assessment of the Knowledge and Perceptions of Precision Medicine (PM) in the Rwandan Healthcare Setting
Source: J Pers Med. 2023 Dec 14;13(12):1707. doi: 10.3390/jpm13121707 (PMC10744509; doi:10.3390/jpm13121707)
Supplement: Supplementary file 1 [file jpm-13-01707-s001.zip › jpm-2584291-supplementary.pdf]

Supplementary data

Table S1: Beliefs of PM implementation in Rwandan healthcare settings.

Key questions	*Interview quotes*
How study participants defined the concept of PM	*"PM is about customization of treatment of a patient depending on her/his lifestyle or depending on his genetic make-up. There are some biomarkers that can be analyzed to orient or to customize a specific treatment depending on the genetic data of the patient."*<br><br>*"…PM is genetic exams they do to assess the genotypes and other genetic characteristics of each cancer patient. But also, the personal and environmental characteristics are key to orient clinical decision especially in cancer treatment depending on their stage."*
Barriers to implement PM program in Rwandan health facilities	***Religious and Cultural beliefs:***<br><br>*"There are religious people who have attitude and beliefs against rules and regimentations of the government, and they accept them as such; for example, some religions do not accept blood transfusion, even if they are about to die…"*<br><br>***Not cost effective:***<br><br>*"Yes, it really depends on the insurance, apart from RAMA, others commercial insurances include (BRITAM, UAP and so on….) most of cancer drugs are bought under the outpatient stream at the cheaper cost, UAP insurance gives you around 2 million; so, paying for these medications with commercial insurance is expensive, at the end, we notice that we have many patients/beneficiaries of RAMA because it's really limitless. Also, I have this fear that PM will only be benefited by a small proportion of the population because the 90% of the population use 'Mutuelle de santé' which does not cover the medication in private pharmacies."*<br><br>*"Copayment (10%) for patients using health insurance: This copayment is not affordable for cancer patients and discourage them for seeking medical services especially those living in extreme poverty."*<br><br>***Concerns of ethical, legal, and social frameworks:***<br><br>*"I am not aware of the policy strictly precising the use of PM but what I know is that the government and its stakeholders emphasize on the use of technology in all fields including medicine. So, without doubt I think that if the PM is promoted in our settings, I think it will be welcomed but so far, I am not aware of any policy particularly talking about PM"*<br><br>***Limited human resources capabilities:***<br><br>*"for human resources, we may need people with expertise in the filed to run PM program in their settings. Currently, we may be having some experts in Rwanda but, in few numbers…"*
Integration of PM program in the existing technology and health system in Rwanda	*"Basing on the current situation with the available infrastructures and technologies, I think we can use PM in our settings especially in referral hospitals. The technology we have might facilitate the initiation of PM program…said by geneticist expert"*<br><br>*"It is possible because we have a good and stable health system in Rwanda, we can even give an evidence of how Rwanda behaved in this time of COVID-19, this shows that we have a good health*

	system in Rwanda. However, we still have some gaps of those advanced genomic sequencing technologies in the hospitals and these gaps will be addressed as we progress."
Perceived changes in government policies to support the implementation of PM in Rwanda	*"….. it is better that PM program be part of health sector strategic plan so that during planning and resource mobilization, PM can also be part of priorities of the health sector … We need to make it integrated in all those documents but also in all plans we are making."*<br><br>*"What we need to do is to reinforce training on PM program. We have the university of Rwanda, we can integrate some degrees and program of PM, but also, we can send our staff abroad to learn about it. Importantly, we also need faculty exchange program between our universities, teaching hospitals and national reference laboratory with other institutions abroad as a way to share experiences in the use of PM concept. That collaboration will open room for exchange of skills and ideas among staff. Maybe it's awareness of this program to the hospitals, to the population who need this service."*
Access to the required infrastructures for PM in Rwandan cancer centers	*"Yeah regarding technology, the National Reference Laboratory (NRL) has the most of equipment necessary to support PM, but there is still gap in supply chain especially reagents to run molecular diagnostics."*
Human resources capabilities in the implementation of PM program	*"First of all, the available physicians, nurses, lab technicians and researchers in the Rwandan settings must be trained about PM concept."*
